# Patient and Health Care Factors Associated With Long-term Diabetes Complications Among Adults With and Without Mental Health and Substance Use Disorders

**DOI:** 10.1001/jamanetworkopen.2019.12060

**Published:** 2019-09-25

**Authors:** Eric M. Schmidt, James Barnes, Cheng Chen, Jodie Trafton, Susan Frayne, Alex H. S. Harris

**Affiliations:** 1 Center for Innovation to Implementation, Veterans Affairs Palo Alto Health Care System, Menlo Park, California; 2 Stanford Health Policy, Stanford University, Stanford, California; 3 Program Evaluation and Resource Center, Office of Mental Health and Suicide Prevention, Veterans Affairs Palo Alto Health Care System, Menlo Park, California; 4 Department of Psychiatry and Behavioral Sciences, Stanford University, Stanford, California; 5 Division of Primary Care and Population Health, Stanford University, Stanford, California; 6 Department of Surgery, Stanford University, Stanford, California; 7 Department of Medicine, Stanford University, Stanford, California

## Abstract

**Importance:**

Among people with diabetes, co-occurring mental health (MH) or substance use (SU) disorders increase the risk of medical complications. Identifying how to effectively promote long-term medical benefits for at-risk populations, such as people with MH or SU disorders, is essential. Knowing more about how health care accessed before the onset of diabetes is associated with health benefits after the onset of diabetes could inform treatment planning and population health management.

**Objective:**

To analyze how preexisting MH or SU disorders and primary care utilization before a new diabetes diagnosis are associated with the long-term severity of diabetes complications.

**Design, Setting, and Participants:**

This cohort study analyzed medical record data from US Department of Veterans Affairs health care systems nationwide and used mixed-effects regressions to test associations between prediabetes patient or health care factors and longitudinal progression of diabetes complication severity from 2006 to 2015. Participants included patients who received a new diabetes diagnosis in 2008 and who were aged 18 to 85 years at the time of their diagnosis. Data analysis was conducted from March to August 2017.

**Exposures:**

Patients were assigned to groups on the basis of a 2-year look-back period for MH or SU disorders status (MH disorder only, SU disorder only, MH and SU disorder, or no MH or SU disorder diagnoses) and on the basis of the amount of primary care utilization before diabetes was diagnosed.

**Main Outcomes and Measures:**

Nine-year trajectories of Diabetes Complication Severity Index (DCSI) scores.

**Results:**

Among 122 992 patients with newly diagnosed diabetes, the mean (SD) age was 62.3 (11.1) years, 118 810 (96.6%) were male, and 28 633 (23.3%) had preexisting MH or SU disorders diagnoses. From the onset of diabetes to 7 years later, patients’ mean estimated DCSI scores increased from 0.84 (95% CI, 0.82-0.87) to 1.42 (95% CI, 1.36-1.47). Controlling for sociodemographic characteristics and medical comorbidities, SU disorders only (decrease in DCSI score, −0.09; 95% CI, −0.13 to −0.04; *P* < .001) or both MH and SU disorders (decrease in DCSI score, −0.13; 95% CI, −0.16 to −0.09; *P* < .001), but not MH disorders only, were associated with lower DCSI scores at the time of the onset of diabetes compared with no MH or SU disorders. More than 90% of patients with MH or SU disorders had primary care visits before diabetes was newly diagnosed, compared with approximately 58% of patients without MH or SU disorders. Patients who had primary care visits before the onset of diabetes had lower baseline DCSI scores, compared with patients who did not have primary care visits (decrease in DCSI score, −0.41 [95% CI, −0.43 to −0.39] for 1-2 visits, −0.50 [95% CI, −0.52 to −0.48] for 3-4 visits, −0.39 [95% CI, −0.41 to −0.37] for 5-8 visits, and −0.15 [95% CI, −0.17 to −0.12] for ≥9 visits; *P* < .001 for all). Patients with MH or SU disorders had lower overall, but more rapidly progressing, mean DCSI scores through year 7 after the onset of diabetes (MH disorder only, 0.006 [95% CI, 0.005-0.008], *P* < .001; SU disorder only, 0.005 [95% CI, 0.001-0.008], *P* = .004; or both MH and SU disorders, 0.008 [95% CI, 0.006-0.011], *P* < .001), compared with patients without MH or SU disorders.

**Conclusions and Relevance:**

Access to and engagement in integrated health care may be associated with modest, albeit impermanent, long-term health benefits for patients with MH and/or SU disorders with newly diagnosed diabetes.

## Key Points

**Question:**

Among patients with newly diagnosed diabetes, are preexisting mental health (MH) or substance use (SU) disorders and primary care utilization before a new diabetes diagnosis associated with the long-term severity of diabetes complications?

**Findings:**

In this cohort study of 122 992 patients in the US Department of Veterans Affairs health system, more than 90% of patients with MH or SU disorders had primary care visits before diabetes was newly diagnosed, compared with approximately 58% of patients without MH or SU disorders. Patients with MH or SU disorders experienced significantly lower severity of diabetes complications for 7 years thereafter, compared with patients without preexisting MH or SU disorders, even after controlling for sociodemographic characteristics and medical comorbidities.

**Meaning:**

Among patients with MH or SU disorders, receiving more medical and mental health care from an integrated health care system before the onset of diabetes is associated with modest, albeit impermanent, health benefits after the onset of diabetes.

## Teaser

This cohort study uses data from the US Veterans Affairs health care systems to examine whether preexisting mental health or substance use disorders and primary care utilization before a new diabetes diagnosis are associated with the long-term severity of diabetes complications.

## Introduction

The global prevalence of diabetes is extensive and increasing.^1^ The duration of living with diabetes is also increasing, prolonging the time during which diabetes complications can occur.^2^ To effectively manage population risks for a chronic disease such as diabetes, it is critical to identify and implement health care delivery models that minimize health-limiting, costly complications that patients experience in the long term.^3,4,5,6,7^

People with diabetes and co-occurring mental health (MH) or substance use (SU) disorders are at increased morbidity and mortality risks, including complications.^8,9,10^ In the context of MH or SU disorders, health care delivery models that integrate medical and behavioral care can reduce the risk of developing diabetes^11,12^ and can help minimize complication risks among patients who develop diabetes.^13^ Access to and engagement in health care before the onset of diabetes may also have substantial health benefits for patients.^14,15^ Primary care is the main hub for diabetes prevention and management in integrated models of care delivery.^16,17^ However, to our knowledge, no previous analyses have examined whether health care received before the onset of diabetes is associated with long-term severity of complications among patients with MH or SU disorders. Knowing more about such long-term associations could inform the design of health care systems that promote integrated care^16,17,18^ and initiatives to improve population health outcomes.^19^

We asked 2 research questions. First, how does complication severity progress differently after a new diabetes diagnosis among patients who do, and do not, have preexisting MH or SU disorder diagnoses? Second, is engagement in primary care before a new diabetes diagnosis associated with long-term severity of diabetes complications? We analyzed 9 years (2006-2015) of national medical record data for patients who received care from the US Department of Veterans Affairs (VA), which operates a large integrated medical and behavioral health care system.

## Methods

### Cohort Selection

Patients with a new diabetes diagnosis were identified through the VA’s Corporate Data Warehouse. Patients were assigned an index date on the basis of their earliest diabetes diagnosis (*International Classification of Diseases*, *Ninth Revision*, *Clinical Modification* codes 250.xx, 357.2, 362.0x, and 366.41) from any inpatient or outpatient health care encounter occurring from October 1, 2007, through September 30, 2008. To be considered a new diabetes diagnosis, no previous diagnoses must have been made for 2 years preceding a patient’s index date (159 754 patients).^20^ Patients were then excluded if they did not receive a second diabetes diagnosis within 2 years after their index date (2329 patients),^20^ were not aged 18 to 85 years on their index date (5385 patients), had missing or irregular sociodemographic data (21 767 patients), had diagnoses in the 2 years before their index date for steroid-induced diabetes or health states that might take precedence over long-term risk management (eg, metastatic cancer, vital organ transplants, severe malnutrition, or dialysis for stage V kidney failure) (4737 patients),^21^ or received most of their VA care outside the United States (2544 patients). Type 2 diabetes was the index diagnosis for 98.6% of patients. This study was approved by the Stanford University institutional review board with a waiver for consent because the data were deidentified. This study follows the Strengthening the Reporting of Observational Studies in Epidemiology (STROBE) reporting guideline.

### Outcome

The validated Diabetes Complication Severity Index (DCSI) was applied to 9 years of inpatient and outpatient medical record data to measure the severity of a patient’s complications.^22^ The DCSI has been used in longitudinal analyses^23^; a 1-point increase in DCSI score is associated with 29% higher risk for hospitalization and 34% higher risk of death within 4 years.^22^ To calculate DCSI scores, we identified diagnoses for 7 categories of complications: cardiovascular complications, cerebrovascular complications, nephropathy, neuropathy, peripheral vascular disease, retinopathy, and metabolic complications. Each complication diagnosis identified was assigned a weight of 1 or 2 points and summed within a category. A patient’s overall DCSI score was calculated as the sum of category totals. Possible DCSI scores range from 0 to 13, with higher scores indicating more-severe complications.^22^ Diabetes Complication Severity Index scores were calculated for the 2-year period before a patient’s index diabetes diagnosis date and annually for each of 7 years thereafter. The DCSI scores were coded as missing during periods when a patient did not use VA care and for each year after a patient was deceased. Data are not assumed to be missing at random (eg, because of death). Patients had a mean (SD) of 6.5 (2.1) points of 8 possible outcome data points. See eFigures 1, 2, 3, and 4 and eTable 1 in the Supplement for complete missingness data.

### Main Comparisons

Patients’ longitudinal trajectories of DCSI scores were compared according to whether they received an MH or SU disorder diagnosis in the 2-year period before their index diabetes diagnosis. *International Classification of Diseases*, *Ninth Revision*, *Clinical Modification* diagnosis codes for MH disorders included bipolar or depressive disorder, posttraumatic stress disorder, anxiety disorder, schizophrenia or other psychoses, or personality disorder (295.xx-298.xx, 300.xx, 301.xx, 307.1, 307.5x, 308.x, 309.xx, 311, 312.xx, and 314.xx). *International Classification of Diseases*, *Ninth Revision*, *Clinical Modification* diagnosis codes for SU disorder included any alcohol, opiate, sedative, cocaine or other stimulant, cannabis, or other drug use disorder (291.xx, 292.xx, 303.xx, 304.xx, 305.0x, and 305.2x-305.9x). Consistent with prior research,^24^ patients were assigned to 4 groups for main comparisons: MH disorders only, SU disorders only, both MH and SU disorders, or neither MH nor SU disorders. Patients without MH or SU disorder diagnoses were the reference group in analyses. See eTable 2 in the Supplement for rates of preexisting MH or SU disorder diagnoses.

We analyzed associations between primary care engagement before the onset of diabetes and baseline DCSI scores. For patients who were engaged in primary care but had no known health conditions before the onset of diabetes, annual primary care visits might be expected in the VA’s version of the patient-centered medical home (ie, 1-2 visits before the onset of diabetes).^17^ Primary care utilization would likely be higher among patients with more clinical complexity (ie, >1 visit per year).^25^ On the basis of a patient’s number of visits to primary care during the 2 years before the onset of diabetes, we assigned patients to each of 5 groups: no visit, 1 to 2 visits, 3 to 4 visits, 5 to 8 visits, or 9 or more visits.

### Covariates

Information about a patient’s age, sex, race/ethnicity, marital status, homeless status, and location of their residence (urban, rural, or highly rural) at the time of the onset of diabetes was obtained from VA’s health record. Medical comorbidities in the 2 years before the onset of diabetes were identified in inpatient and outpatient data using the Elixhauser Comorbidity Index and were coded into 15 dummy variables,^26^ excluding those comorbidities that overlapped with our outcome variable (eg, nephropathy) or a main comparison by MH or SU disorders (eg, alcohol use disorder). Laboratory and medication data were not included because they were not available before the onset of diabetes, and their inclusion after the onset of diabetes would overcontrol the analyses.

### Statistical Analysis

 Data analysis was conducted from March to August 2017. After we calculated descriptive statistics, a test of independent proportions was used to determine whether the percentage of diabetes complications differed significantly by MH or SU disorder status over all periods. A mixed-effects linear regression was used to test associations between MH or SU disorder status and 3 parameters estimated to describe the longitudinal trajectories of DCSI scores: intercept term, linear term, and quadratic term. Associations between primary care utilization and longitudinal DCSI scores were estimated for effects on the intercept term, which signifies the association of medical care engagement with baseline DCSI scores. To account for the clustered nature of these data, we used a 3-level model to nest the annual DCSI scores first within patients and then for patients within 1 of 138 VA health care systems where each patient received the most primary care before their index diabetes diagnosis. Random effects for each patient’s intercept and slope of DCSI scores were modeled with an autoregressive covariance structure for repeated measures and an exchangeable covariance structure for the clustering of patients within VA health care systems. Including a quadratic term for time, in addition to the linear term, allowed the estimated DCSI trajectories to curve, which provided both superior statistical and theoretical fit (ie, severity of complications may not progress linearly over time). Regression model building followed 3 steps: an unconditional longitudinal growth model, a longitudinal growth model conditional only on MH or SU disorder status, and a conditional longitudinal growth model with primary care utilization and fully adjusted for covariates described already. A significance level of α = .05, based on a 2-sided Wald test, was used throughout. Regression analyses were conducted using the LME function in the NLME library of the R statistical program version 3.4.0 (R Project for Statistical Computing).^27,28^

We conducted 2 sensitivity analyses. First, given the higher rates of mortality in the population with MH or SU disorders^29,30,31^ and positive associations between a DCSI score and mortality,^22^ we were concerned about bias due to fewer, yet apparently more-severe, outcome data points over time in the MH or SU disorders groups. Therefore, we reran the fully adjusted regression analysis limited to patients who were alive through year 7 of the outcome period, excluding patients whose outcome data were missing because of death. Second, associations between primary care utilization and complication trajectories could be affected by patients who did not have primary care visits or who received primary care elsewhere before the onset of diabetes. Therefore, we ran a second sensitivity analysis limited to those patients who had VA primary care visits at least twice in the 2 years before the onset of diabetes.

## Results

Among 122 992 VA patients with newly diagnosed diabetes, 28 633 (23.3%) had an MH and/or SU disorder diagnosis in the 2 years before their index diabetes diagnosis date (Table 1). The mean (SD) age of the cohort was 62.3 (11.1) years, and 118 810 (96.6%) were male. Patients with an MH or SU disorder diagnosis were younger (mean [SD] ages: MH only, 60.2 [10.8], years; SU only, 57.5 [9.1] years; both MH and SU, 55.7 [8.3] years) than patients without MH or SU disorders diagnoses (mean [SD] age, 63.3 [11.1] years). Substance use disorder diagnoses were more common among male (99.0%), black (35.7%), unmarried (69.3%), and homeless (15.3%) patients. Patients with MH or SU disorders had higher prediabetes rates of all medical comorbidities analyzed; in particular, hypertension was diagnosed twice as often in patients with than in those without MH or SU disorders before the onset of diabetes (MH only, 66.5%; SU only 63.4%; both MH and SU, 65.7%; no MH or SU, 33.9%). Hypertension diagnosis rates were similar across groups in the year after the onset of diabetes (MH only, 73.1%; SU only, 73.9%; both MH and SU, 72.1%; no MH or SU, 76.3%). More than 90% of patients with MH or SU disorders had primary care visits in the 2 years before their first diabetes diagnosis, compared with approximately 58% of patients without MH or SU disorders. Approximately 98% of all patients had primary care visits during the first year after their new diabetes diagnosis, and a higher proportion of patients with MH or SU disorders vs those without MH or SU disorders had primary care visits each year thereafter (eg, for patients with both MH and SU disorders vs patients with no MH or SU disorders, year 2, 94.5% vs 85.7%; year 3, 92.7% vs 83.7%; year 4, 97.1% vs 82.1%; year 5, 90.4% vs 80.8%; year 6, 89.4% vs 79.9%; year 7, 88.7% vs 66.9%) (Table 1).

**Table 1.** Demographic Characteristics of Cohort of Veterans With Diabetes, by MH and SU Disorder Group

Characteristic	MH Disorder Only (n = 20 032)	SU Disorder Only (n = 2800)	Both MH and SU (n = 5801)	Neither MH nor SU (n = 94 359)	Total (N = 122 992)
Age, mean (SD), y^a^	60.2 (10.8)	57.5 (9.1)	55.7 (8.3)	63.3 (11.1)	62.3 (11.1)
Male, %	93.3	99.0	95.7	97.3	96.6
Race/ethnicity, %					
White	76.7	57.0	61.7	75.5	74.6
Black	16.0	35.7	30.2	16.9	17.8
Hispanic or Latino	5.3	5.0	6.0	5.0	5.1
Asian	1.3	1.1	1.2	1.8	1.7
American Indian	0.8	1.2	0.9	0.8	0.8
Married, %^a^	55.3	30.7	34.6	61.8	58.7
Homeless, %^b^	5.3	15.3	23.1	1.7	3.2
Died, %^c^	15.4	20.7	17.2	16.8	16.7
Location of residence, %^a^					
Highly rural	1.2	1.5	1.1	1.3	1.3
Rural	38.5	27.6	27.7	36.2	36.0
Urban	60.3	70.9	71.3	62.5	62.7
Inpatient index diabetes diagnosis, %	5.1	11.1	11.2	3.3	4.2
Medical inpatient stay, %^b^	15.0	21.4	37.7	4.0	7.8
Primary care visits before diabetes diagnosis, %^b^					
0 Visit	4.7	10.1	4.5	41.8	33.2
1-2 Visits	12.9	17.8	9.7	22.0	19.8
3-4 Visits	15.2	16.6	11.8	11.9	12.5
5-8 Visits	29.8	27.8	26.4	14.6	17.9
≥9 Visits	37.4	27.8	47.6	9.9	16.6
Had primary care visit, by year after new diabetes diagnosis, %^d^					
Year 1	98.2	97.1	98.2	98.1	98.1
Year 2	94.1	91.3	94.5	85.7	87.6
Year 3	92.3	88.6	92.7	83.7	85.6
Year 4	91.3	87.9	91.7	82.1	84.2
Year 5	90.2	86.9	90.4	80.8	82.9
Year 6	88.8	85.8	89.4	79.9	81.9
Year 7	88.0	84.9	88.7	66.9	81.2
Diagnosed comorbidities, %^b^					
Pulmonary circulation disorder	0.7	0.6	0.5	0.2	0.3
Hypertension	66.5	63.4	65.7	33.9	41.4
Paralysis	1.3	0.9	1.2	0.4	0.6
Other neurologic disorder	5.0	2.8	6.0	1.1	2.0
Chronic obstructive pulmonary disease	22.5	19.9	26.1	8.2	11.7
Hypothyroidism	7.2	3.2	4.9	2.8	3.7
Liver disease	2.9	10.8	11.6	0.8	1.9
Chronic peptic ulcer	2.1	1.8	2.5	0.7	1.0
Solid tumor without metastases	10.5	8.1	7.5	5.5	6.5
Rheumatoid arthritis	2.2	1.1	1.5	0.9	1.1
Coagulopathy	1.9	2.4	2.8	0.9	1.2
Fluid and electrolyte disorders	4.1	5.0	6.7	1.3	2.1
Blood loss anemia	0.1	0.2	0.2	<0.1	<0.1
Deficiency anemia	6.3	7.1	8.1	2.4	3.4
Obesity	32.9	25.0	30.0	13.0	17.3
Tobacco use disorder	22.1	43.7	50.0	7.1	12.4

Abbreviations: MH, mental health; SU, substance use.

^a^ On the date of index diabetes diagnosis.

^b^ During the 2-year period before the index diabetes diagnosis date.

^c^ Death during the 7-year observation period after the date of initial diabetes diagnosis.

^d^ See eFigure 2 in the Supplement for the numbers of patients used to calculate the percentages.

Seven years after their first diabetes diagnosis, compared with patients without MH or SU disorders, patients in any MH or SU disorders group had higher diagnosed rates of neuropathy (MH only, 44.0%; SU only, 38.3%; both MH and SU, 44.8%; no MH or SU, 34.4%), but lower rates of retinopathy (MH only, 25.5%; SU only, 28.6%; both MH and SU, 26.5%; no MH or SU, 30.3%) and nephropathy (MH only, 16.5%; SU only, 15.9%; MH and SU, 15.7%; no MH or SU, 20.3%) (Table 2). Patients with SU disorders alone or with both MH and SU disorders experienced lower rates of cardiovascular complications (44.1% and 43.9%, respectively), but higher rates of peripheral vascular disease (21.6% and 19.5%, respectively) and metabolic complications (2.9% and 2.0%, respectively) compared with patients with MH disorders only (cardiovascular complications, 49.7%; peripheral vascular disease, 16.6%; metabolic complications, 0.9%) and no MH or SU disorders (cardiovascular complications, 49.6%; peripheral vascular disease, 16.9%; metabolic complications, 0.9%). Cerebrovascular complications were highest among patients with MH diagnosis only (17.4%). See eTable 3 in the Supplement for example diagnoses included in the DCSI and eTable 4 in the Supplement for complication event rates by comparative groups across all periods analyzed.

**Table 2.** Distribution of Diabetes Complications Over the Course of 9 Years, by MH or SU Disorder Group^a^

Diabetes Complication Category	Weight^b^	MH Disorder Only (n = 20 032)	SU Disorder Only (n = 2800)	MH and SU Disorder (n = 5801)	No MH or SU Disorder (n = 94 359)	Total (N = 122 992)
Cardiovascular, %						
Any		49.7	44.1	43.9	49.6	49.2
Mild	1	41.4	33.6	35.0	40.9	40.5
Severe	2	28.7	28.0	26.7	28.4	28.4
Neuropathy, %	1	44.0	38.3	44.8	34.4	36.6
Retinopathy, %						
Any		25.5	28.6	26.5	30.3	29.3
Mild	1	17.8	22.2	18.1	24.6	23.2
Severe	2	10.7	10.5	11.2	10.7	10.7
Nephropathy, %						
Any		16.5	15.9	15.7	20.3	19.4
Mild	1	16.5	15.9	15.7	20.3	19.4
Severe	2	14.5	14.1	13.6	17.9	17.0
Peripheral vascular disease, %						
Any		16.6	21.6	19.5	16.9	17.1
Mild	1	13.7	17.5	15.5	13.9	14.1
Severe	2	5.2	9.2	7.4	6.1	6.1
Cerebrovascular, %						
Any		17.4	15.7	15.1	14.7	15.2
Mild	1	5.1	3.4	4.3	3.4	3.7
Severe	2	14.9	14.0	13.1	13.0	13.3
Metabolic, %	2	0.9	2.9	2.0	0.9	1.0

Abbreviations: MH, mental health; SU, substance use.

^a^ Note that the 9-year period of data analyzed for complications includes 2 years before a patient’s new diabetes diagnosis and 7 years of follow-up. Tests of independent proportions were used to identify significant differences in proportion of complications across mental and substance use disorder categories (*P* < .001 for all complication categories).

^b^ The weight listed is how much a diagnosis from each complication category contributes to a patient’s overall Diabetes Complication Severity Index score.

### Mixed-Effects Regression Estimating Longitudinal Complication Severity

The unconditional model estimating longitudinal complication severity indicated that patients started with a mean estimated DCSI score of 0.84 (95% CI, 0.82-0.87) (ie, approximately 1 mild complication) at the time of their first diabetes diagnosis and had a mean estimated DCSI score of 1.42 (95% CI, 1.36-1.47) 7 years later (Table 3, model A).

**Table 3.** Unstandardized Estimates From Mixed-Effects Regression Estimating Longitudinal Diabetes Complication Severity Index Scores^a^

Variable	Model A, Unconditional		Model B, Conditional, Unadjusted		Model C, Conditional, Adjusted^b^	
	Estimate (95% CI)	*P* Value	Estimate (95% CI)	*P* Value	Estimate (95% CI)	*P* Value
Intercept	0.84 (0.82 to 0.87)	<.001	0.84 (0.82 to 0.86)	<.001	−0.31 (−0.35 to −0.27)	<.001
MH or SU disorder status (reference: none)						
MH disorder only	NA	NA	0.01 (−0.01 to 0.03)	.32	0.02 (−0.004 to 0.03)	.12
SU disorder only	NA	NA	−0.08 (−0.13 to −0.04)	.001	−0.09 (−0.13 to −0.04)	<.001
MH and SU disorder	NA	NA	−0.04 (−0.07 to −0.01)	.01	−0.13 (−0.16 to −0.09)	<.001
Time, y	0.08 (0.08 to 0.09)	<.001	0.11 (0.10 to 0.11)	<.001	0.10 (0.10 to 0.11)	<.001
Time, y^2^	−0.002 (−0.002 to −0.001)	<.001	−0.004 (−0.004 to −0.003)	<.001	−0.003 (−0.004 to −0.003)	<.001
Interaction: time × MH or SU disorder (reference: none)						
MH disorder only	NA	NA	−0.08 (−0.09 to −0.07)	<.001	−0.07 (−0.08 to −0.06)	<.001
SU disorder only	NA	NA	−0.05 (−0.07 to −0.03)	<.001	−0.04 (−0.07 to −0.02)	<.001
MH and SU disorder	NA	NA	−0.10 (−0.12 to −0.08)	<.001	−0.09 (−0.11 to −0.08)	<.001
Interaction: time^2^ × MH or SU disorder (reference: none)						
MH disorder only	NA	NA	0.007 (0.006 to 0.008)	<.001	0.006 (0.005 to 0.008)	<.001
SU disorder only	NA	NA	0.005 (0.002 to 0.008)	.001	0.005 (0.001 to 0.008)	.004
MH and SU disorder	NA	NA	0.009 (0.007 to 0.011)	<.001	0.008 (0.006 to 0.011)	<.001
Primary care visits, before diabetes diagnosis (reference: no visit)						
1-2 Visits	NA	NA	NA	NA	−0.41 (−0.43 to −0.39)	<.001
3-4 Visits	NA	NA	NA	NA	−0.50 (−0.52 to −0.48)	<.001
5-8 Visits	NA	NA	NA	NA	−0.39 (−0.41 to −0.37)	<.001
≥9 Visits	NA	NA	NA	NA	−0.15 (−0.17 to −0.12)	<.001

Abbreviations: MH, mental health; NA, not applicable; SU, substance use.

^a^ Unstandardized estimates are in units of a Diabetes Complication Severity Index score, with a 1-point difference representing approximately 1 mild-severity diabetes complication or the difference between a mild and severe complication (ie, a higher score indicates a higher overall severity of diabetes complications).

^b^ See eTable 5 in the Supplement for unstandardized estimates of variables used to adjust multivariable model C. Model A was an unconditional mixed-effects regression model. Model B was conditional on the MH or SU disorder diagnosis status of patients in the 2-year period before their index diabetes diagnosis date. Model C was conditional on the MH or SU disorder status of patients in the 2-year period before their index diabetes diagnosis date and adjusted for age, race/ethnicity, marital status, homelessness status, location of residence (urban vs rural or highly rural), medical comorbidities, and primary care and inpatient medical care utilization.

After adding MH or SU disorders to the model (Table 3, model B), SU only (−0.08; 95% CI, −0.13 to −0.04; *P* = .001) and both MH and SU (−0.04; 95% CI, −0.07 to −0.01; *P* = .01), but not MH only, were associated with significantly lower intercept terms (ie, less-severe baseline DCSI scores when diabetes was first diagnosed). Compared with patients without MH or SU disorders, having any MH or SU disorder was associated with significantly less severely progressing slope terms for a patient’s DCSI score after diabetes was diagnosed (MH only, −0.08 [95% CI, −0.09 to −0.07], *P* < .001; SU only, −0.05 [95% CI −0.07 to −0.03], *P* < .001; both MH and SU, −0.10 [95% CI, −0.12 to −0.08], *P* < .001). However, compared with patients without MH or SU disorder, having any MH or SU disorder was associated with a significantly higher quadratic term from year to year, which indicated more rapidly increasing DCSI scores among patients with MH or SU disorders as more time passed since diabetes was diagnosed (MH only, 0.007 [95% CI, 0.006-0.008], *P* < .001; SU only, 0.005 [95% CI, 0.002-0.008], *P* = .001; or both MH and SU, 0.009 [95% CI, 0.007-0.011], *P* < .001).

Adjusting for demographic characteristics and medical comorbidities did not substantively alter associations between the presence of MH or SU disorder and DCSI score trajectories. Controlling for sociodemographic characteristics and medical comorbidities, SU only (−0.09; 95% CI, −0.13 to −0.04; *P* < .001) or both MH and SU (−0.13; 95% CI, −0.16 to −0.09; *P* < .001), but not MH only, were associated with lower mean DCSI scores at the time of the onset of diabetes compared with no MH or SU disorders. Patients with MH or SU disorders had lower overall, but more rapidly progressing, mean DCSI scores through year 7 after the onset of diabetes (MH only, 0.006 [95% CI, 0.005-0.008], *P* < .001; SU only, 0.005 [95% CI, 0.001-0.008], *P* = .004; or both MH and SU, 0.008 [95% CI, 0.006-0.011], *P* < .001), compared with patients without MH or SU disorders. Compared with patients who did not have primary care visits in the 2 years before diabetes was first diagnosed, higher primary care utilization in any amount was associated with lower mean baseline DCSI scores (−0.41 [95% CI, −0.43 to −0.39] for 1-2 visits, −0.50 [95% CI, −0.52 to −0.48] for 3-4 visits, −0.39 [95% CI, −0.41 to −0.37] for 5-8 visits, and −0.15 [95% CI, −0.17 to −0.12] for ≥9 visits; *P* < .001 for all) (Table 3, model C; eTable 5 in the Supplement).

On the basis of the fully adjusted regression estimates, the Figure displays estimated DCSI trajectories over time, by MH or SU disorder group, with an extrapolation to 13 years after a patient’s new diabetes diagnosis (assuming that regression estimates hold beyond the 7 years we analyzed directly). As seen in the Figure, although overall DCSI scores were less severe 7 years after a new diabetes diagnosis for patients with MH or SU disorders compared with those without such disorders (eg, patients with both MH and SU disorders had a DCSI score one-third of a point lower than did patients without MH or SU disorders), the direction of this difference was estimated to reverse 10 to 12 years after a new diabetes diagnosis.

**Figure.**
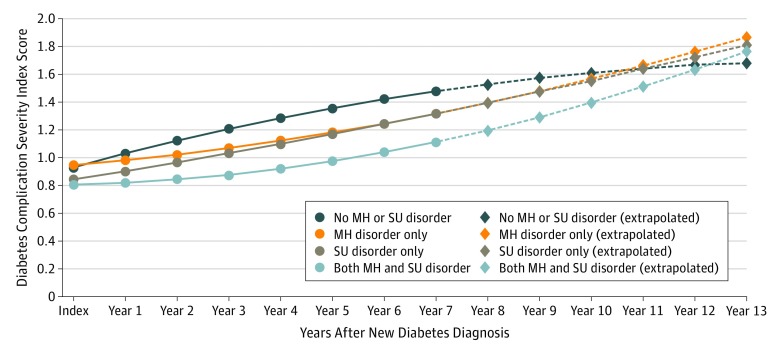
Estimated and Extrapolated Trajectories of Diabetes Complication Severity Index (DCSI) Scores After a New Diabetes Diagnosis Estimated longitudinal trajectories of DCSI score are shown by mental health (MH) or substance use (SU) disorder status, after adjusting for sociodemographic characteristics and health care utilization (ie, model C, Table 3; eTable 5 in the Supplement). Estimated trajectories were calculated for an illustrative composite patient whose index diabetes diagnosis was made at age 60 years. Circles and solid lines represent DCSI score trajectories, where DCSI ranges from 0 to 13 and a higher DCSI score indicates more-severe diabetes complications, based on observed data. Diamonds and dashed lines represent extrapolated estimated trajectories beyond the end of this study’s observation period to illustrate the implications of estimated trajectories 13 years after a new diabetes diagnosis. Although DCSI scores are more favorable for patients with MH or SU disorders than for patients without MH or SU disorders in the early years after a new diabetes diagnosis, this association is estimated to reverse within 10 to 12 years after diabetes is first diagnosed.

### Sensitivity Analyses

In a sensitivity analysis limited to patients who remained alive through year 7 after diabetes was first diagnosed, associations between the presence of MH or SU disorders and longitudinal DCSI scores and those between primary care utilization and DCSI scores remained significant and were generally similar to the main regression model (eTable 6 in the Supplement). In the sensitivity analysis limited to patients who had 2 or more primary care visits before their first diabetes diagnosis, MH only was associated with significantly higher mean initial DCSI score (0.05; 95% CI, 0.03-0.07; *P* < .001), having both MH and SU before diabetes was associated with significantly lower initial mean DCSI score (−0.09; 95% CI, −0.12 to −0.05; *P* < .001), and SU only was not associated with a different initial score compared with patients without MH or SU disorders. However, the ways in which DCSI trajectories progressed over time were similar in terms of magnitude of increase or curve compared with the main analysis. In the second sensitivity analysis, compared with patients with 2 primary care visits before diabetes was newly diagnosed, 3 to 4 visits were associated with significantly lower mean DCSI scores (−0.08; 95% CI −0.10 to −0.05; *P* < .001), 5 to 8 visits were not associated with different mean DCSI scores, and 9 or more visits were associated with significantly higher mean DCSI scores (0.24; 95% CI 0.21 to 0.26; *P* < .001) (eTable 6 in the Supplement). The ways that DCSI trajectories progressed in the second sensitivity analysis were similar compared with the main analysis.

## Discussion

This study is the first, to our knowledge, to compare longitudinal severity of multiple diabetes complications simultaneously among patients with newly diagnosed diabetes who do and do not have MH or SU disorders. Our analyses suggest that, for patients with MH or SU disorders, receiving health care from the VA’s integrated medical and behavioral health care system before the onset of diabetes might have long-term, albeit impermanent, protective effects against the onset or worsening of diabetes complications. Our results are surprising given that MH or SU disorders can interact with self-management demands and treatment processes, increasing risk for diabetes-related morbidity and mortality.^9,32,33,34,35^

We found statistically significant associations between having an MH or SU disorder diagnosis before diabetes was first diagnosed and lower overall severity of diabetes complications thereafter in a national cohort of VA patients. However, complication severity progressed differently from year to year between patients with and without MH or SU disorders. We estimated that patients with MH or SU disorders would experience more-severe complications approximately 10 to 12 years after diabetes was diagnosed. Associations between MH or SU status and lower complication severity could be related to having received treatment for an MH or SU disorder before the onset of diabetes. It seems plausible that receiving effective treatment for MH or SU disorder symptoms before the onset of diabetes could account for why we did not find higher severity of complications among the patients with MH or SU disorders in our study. However, it is less plausible that MH or SU disorder treatment alone, received before diabetes was diagnosed, would account for the significantly lower severity of diabetes complications we observed, compared with patients without MH or SU disorder diagnoses.

Another potential explanation for our findings might lie in patients’ primary care engagement. The most prevalent MH or SU disorder diagnoses in our cohort were depression and alcohol use disorders, both of which can be monitored and treated effectively in primary care.^36,37^ Compared with 58% of patients without MH or SU disorders, more than 90% of patients with MH or SU disorders had primary care visits in the 2-year period before the onset of diabetes, which likely reflects greater clinical complexity.^38^ In our study, diabetes complications were modestly less severe among patients who had primary care visits before the onset of diabetes, indicating the importance of access to and engagement in primary care in mitigating complication severity. Although we cannot draw causal connections on the basis of the current data, consider hypertension, which is important to manage in the context of diabetes, as an example of how primary care might be associated with complications in the population with MH or SU disorders.^39^ Hypertension was diagnosed twice as often in patients with than in those without MH or SU disorders before the onset of diabetes, but hypertension rates were similar across groups the subsequent year. Therefore, more patients with MH or SU disorders were likely already managing blood pressure at the onset of diabetes, even though a similar proportion of patients without MH or SU disorders could have benefited from blood pressure management before the onset of diabetes (ie, hypertension was undetected). Perhaps patients with MH or SU disorders had more clinical momentum to implement recommended complication prevention practices into their medical care plans at the time of the onset of diabetes.^39^ This explanation is speculative, but clinical momentum, if present, might have been garnered via treatment for preexisting medical conditions, behavioral conditions, or both.^13,40,41,42^

Clinically, our findings may represent modest differences for patients, but important differences at the population level. After 7 years, patients with both MH and SU disorders had a DCSI score one-third of a point lower than did patients without MH or SU disorders. This difference translates into one-third less of a diabetes complication classified as a mild diagnosis per the DCSI (eg, gastroparesis), or one-third less progression from a mild to a severe complication (eg, from diabetes nephropathy to chronic renal failure).^22^ Diabetes Complication Severity Index scores may be driven by higher-prevalence complications (eg, cardiovascular), of which diabetes is but one contributor.^43,44^ Nonetheless, a score on the DCSI is associated with risk for future hospitalization and death^22^; preventing complications by a significant amount on average still represents notable gains in population health given the extreme prevalence and increasing incidence of diabetes.^1,2^

The VA operates a very large integrated health care system, with a version of the patient-centered medical home.^17,45,46^ Patients who had MH or SU disorders in our study had higher medical care utilization rates than did patients without MH or SU disorders, in addition to receiving behavioral health care in the same system, which reflects the VA’s medical-behavioral care integration. Administrative aspects of the VA that promote integration include a shared medical record, direct consultation and referral across service lines, and veterans’ lifelong and continuous enrollment as beneficiaries (ie, there are fewer financial barriers to integration).^17,45,46,47^ Clinically, interdisciplinary clinicians at the VA are often colocated or collaborate across the full range of subspecialty care offered for medical and behavioral conditions.^45,46^ At the VA and elsewhere, policy makers, payers, and organizations that deliver health care, such as those involved with the Centers for Medicare & Medicaid Services’ Shared Savings Program, aim for long-term accountability to population health.^19^ Health care systems that promote integration between medical and behavioral service lines can effectively promote diabetes prevention in the context of co-occurring and interacting health conditions such as MH or SU disorders.^11,12,13^ Our results lend support for designing health care systems to promote access to and engagement in integrated care that, when provided before the onset of a chronic disease, could have long-term health benefits even for patients who ultimately develop a disease such as diabetes.^14,15^ That health care provided before the onset of diabetes might have long-term health benefits could point toward important future research directions. Future research should delineate health care system–level factors that promote engagement in integrated health care that is associated with complication trajectories. It appears worthwhile to investigate the plausibility of clinical momentum to reduce complication risks in patients with MH or SU disorders. How disease control (eg, glycated hemoglobin level) or medication adherence mediate complication trajectories after the onset of diabetes should be examined. Finally, future studies should investigate whether our findings apply equally to all diabetes complications and MH or SU disorders diagnoses.

### Limitations

This study has limitations, notably the role of missing data. Because our outcome variable is positively associated with death,^22^ bias (eg, higher mortality among patients with SU only) could lead to artificially higher initial DCSI scores, faster-increasing DCSI scores, or differently curving DCSI score trajectories. However, associations between MH or SU disorders and DCSI score trajectories were similar in a sensitivity analysis limited to only patients who had no missing outcome data due to death. We coded data as missing each year a patient received no health care from the VA. Patients could have used health care outside the VA that would not be observed in our analysis, which could lead to mischaracterization of patients’ complications. Therefore, we conducted a sensitivity analysis limited to patients who had at least 2 primary care visits before the onset of diabetes. The way complication trajectories progressed over time in the second sensitivity analysis did not change substantively from the main analysis. Our study also had numerous strengths, including the use of data from a large national cohort without gaps in enrollment or eligibility for care, a long observation period, starting the observation period at the time of a new diabetes diagnosis, and rigorous statistical analyses.

## Conclusions

For people with MH or SU disorders who develop diabetes, access to and engagement in an integrated model of health care delivery that includes primary care before the onset of diabetes provides modest, albeit impermanent, long-term health benefits in terms of diabetes complications. For patients with MH or SU disorders, sustained attention to chronic disease management takes on increasing importance as more time passes after the onset of diabetes, particularly because clinicians are likely to be juggling multiple comorbidities simultaneously in these patients.^48^

## Notes

Supplement. **eFigure 1.** Overall Missingness for Outcome Data Points in the Main Analysis **eFigure 2.** Missingness for Outcome Data Points Due to Mortality in the Main Analysis **eFigure 3.** Missingness for Outcome Data Points Due to Not Using VHA Health Care in a Given Time Period, Among Patients Who Were Alive Into Year 7 After Diabetes Onset (Sensitivity Analysis 1) **eFigure 4.** Missingness for Outcome Data Points Due to Mortality or to Not Using VHA Health Care in a Given Time Period, Among Patients Who Made at Least 2 Visits to Primary Care Before New Onset Diabetes (Sensitivity Analysis 2) **eTable 1.** Percent Distribution of Number of Outcome Data Points Analyzed per Patient Across Eight Total Time Periods Possible **eTable 2.** Percent of Patients with Pre-Existing Mental Health or Substance Use Disorder Diagnoses **eTable 3.** Example Diagnoses Included in the Diabetes Complication Severity Index, by Category of Complications **eTable 4.** Percent of Patients Diagnosed With a Specific Category of Diabetes Complications, by Severity of Complication, by Time Period, and by Comparative MHSU Groups **eTable 5.** Complete Mixed Effects Regression Model Results With All Covariates **eTable 6.** Complete Mixed Effects Regression Model Results From Sensitivity Analyses **eReference**
