# Supplementary material for: Patient and Health Care Factors Associated With Long-term Diabetes Complications Among Adults With and Without Mental Health and Substance Use Disorders
Source: JAMA Netw Open. 2019 Sep 25;2(9):e1912060. doi: 10.1001/jamanetworkopen.2019.12060 (PMC6763972; doi:10.1001/jamanetworkopen.2019.12060)
Supplement: Supplement. — eFigure 1. Overall Missingness for Outcome Data Points in the Main Analysis eFigure 2. Missingness for Outcome Data Points Due to Mortality in the Main Analysis eFigure 3. Missingness for Outcome Data Points Due to Not Using VHA Health Care in a Given Time Period, Among Patients Who Were Alive Into Year 7 After Diabetes Onset (Sensitivity Analysis 1) eFigure 4. Missingness for Outcome Data Points Due to Mortality or to Not Using VHA Health Care in a Given Time Period, Among Patients Who Made at Least 2 Visits to Primary Care Before New Onset Diabetes (Sensitivity Analysis 2) eTable 1. Percent Distribution of Number of Outcome Data Points Analyzed per Patient Across Eight Total Time Periods Possible eTable 2. Percent of Patients with Pre-Existing Mental Health or Substance Use Disorder Diagnoses eTable 3. Example Diagnoses Included in the Diabetes Complication Severity Index, by Category of Complications eTable 4. Percent of Patients Diagnosed With a Specific Category of Diabetes Complications, by Severity of Complication, by Time Period, and by Comparative MHSU Groups eTable 5. Complete Mixed Effects Regression Model Results With All Covariates eTable 6. Complete Mixed Effects Regression Model Results From Sensitivity Analyses eReference [file jamanetwopen-2-e1912060-s001.pdf]

Supplementary Online Content

Schmidt EM, Barnes J, Chen C, Trafton J, Frayne S, Harris AHS. Patient and health care factors associated with long-term diabetes complications among adults with and without mental health and substance use disorders. *JAMA Netw Open*. 2019;2(9):e1912060. doi:10.1001/jamanetworkopen.2019.12060

eFigure 1. Overall Missingness for Outcome Data Points in the Main Analysis

eFigure 2. Missingness for Outcome Data Points Due to Mortality in the Main Analysis

eFigure 3. Missingness for Outcome Data Points Due to Not Using VHA Health Care in a Given Time Period, Among Patients Who Were Alive Into Year 7 After Diabetes Onset (Sensitivity Analysis 1)

eFigure 4. Missingness for Outcome Data Points Due to Mortality or to Not Using VHA Health Care in a Given Time Period, Among Patients Who Made at Least 2 Visits to Primary Care Before New Onset Diabetes (Sensitivity Analysis 2)

eTable 1. Percent Distribution of Number of Outcome Data Points Analyzed per Patient Across Eight Total Time Periods Possible

eTable 2. Percent of Patients with Pre-Existing Mental Health or Substance Use Disorder Diagnoses

eTable 3. Example Diagnoses Included in the Diabetes Complication Severity Index, by Category of Complications

eTable 4. Percent of Patients Diagnosed With a Specific Category of Diabetes Complications, by Severity of Complication, by Time Period, and by Comparative MHSU Groups

eTable 5. Complete Mixed Effects Regression Model Results With All Covariates

eTable 6. Complete Mixed Effects Regression Model Results From Sensitivity Analyses

eReference

This supplementary material has been provided by the authors to give readers additional information about their work.

© 2019 Schmidt EM et al. *JAMA Network Open.*

Complete Data on Missing Data Points in the Outcome Variable

This section of the supplement provides data on missing data points over 8 time periods analyzed in the outcome variable using mixed-effects regression models (main analysis and sensitivity analyses). Two sub-sections on missingness are included. First, we present level of missingness of data points, by year and by comparative groups, in the outcome variable (a score on the Diabetes Complication Severity Index[1]). Note that patients in our cohort were enrolled in and eligible for health care throughout the entire study (i.e., no enrollment gaps). Many patients had outcome data points (e.g., in Years 3-7 after new diabetes diagnosis) after having a missing data point during an earlier year of their outcome data (e.g., missing in Year 2). Therefore, second, we present data using an indicator of completeness in our outcome variable over time: the number of outcome data points analyzed per patient.

As described in the Method section of the manuscript, we coded data as missing for either of two reasons:

(1) Mortality – data were coded as missing for each year after a patient's date of death.
(2) Patient who were alive but did not receive health care at Veterans Health Administration (VHA) during a given time period. Note that patients could have received VHA care in a subsequent year, if data were coded as missing due to no VHA care received.

Main Analysis: Bar Charts Displaying Missingness in the Outcome Variable by Year and by Comparative Group

eFigure 1 displays overall missingness in the main analysis (see also Table 3, manuscript). Patients in the No MH or SU group had the most missing data, followed by patients in the SU Only group. Patient with MH had similar levels of missingness, with or without co-occurring SU disorders.

eFigure 1. Overall Missingness for Outcome Data Points in the Main Analysis

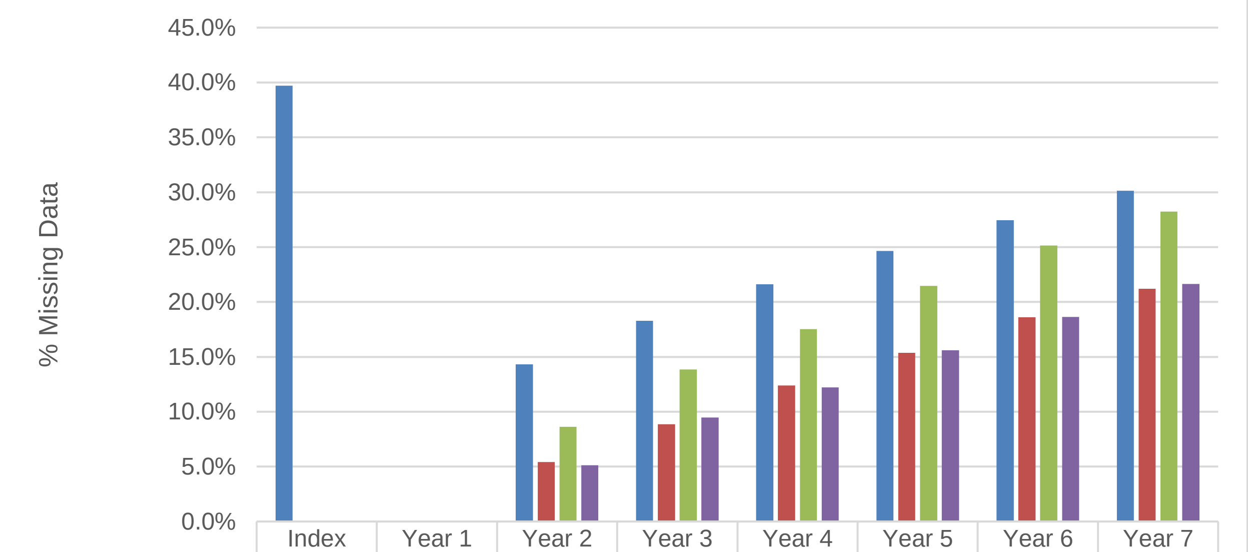

	Index	Year 1	Year 2	Year 3	Year 4	Year 5	Year 6	Year 7
No MH or SU	39.7%	0.0%	14.3%	18.3%	21.6%	24.6%	27.5%	30.1%
MH Only	0.0%	0.0%	5.4%	8.9%	12.4%	15.4%	18.6%	21.2%
SU Only	0.0%	0.0%	8.6%	13.9%	17.5%	21.5%	25.1%	28.3%
Both MH and SU	0.0%	0.0%	5.1%	9.5%	12.2%	15.6%	18.6%	21.6%

© 2019 Schmidt EM et al. *JAMA Network Open.*

eFigure 2 displays the amount of missingness due to mortality among patients included in the main analysis. Patients in the SU Only group had the highest rates of mortality in every time period, starting in Year 2. Note that only patients alive into year 1 were included in analyses.

eFigure 2. Missingness for Outcome Data Points Due to Mortality in the Main Analysis

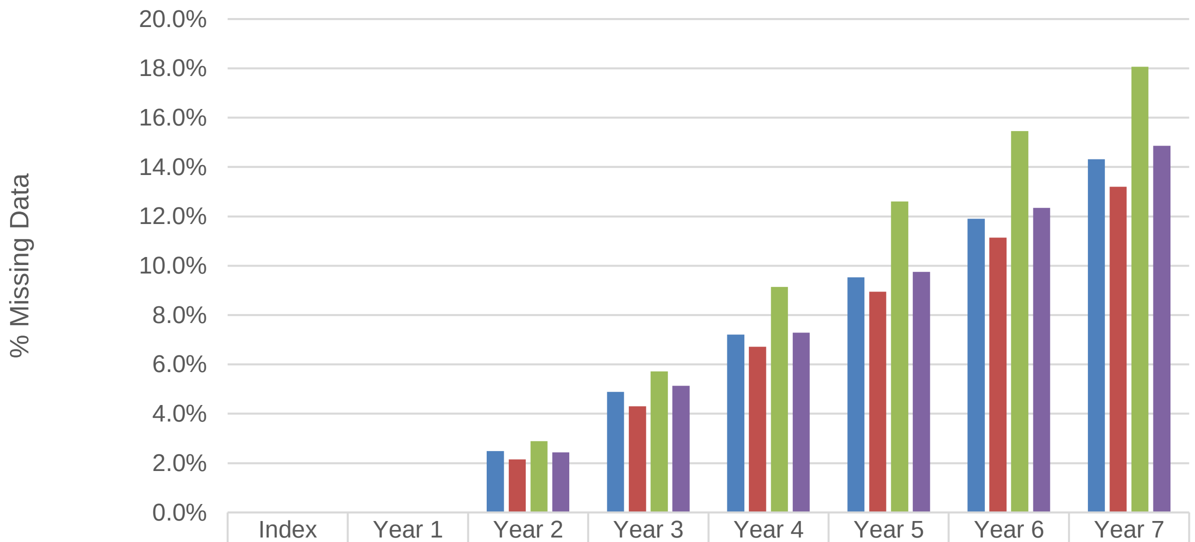

	Index	Year 1	Year 2	Year 3	Year 4	Year 5	Year 6	Year 7
No MH or SU	0.0%	0.0%	2.5%	4.9%	7.2%	9.5%	11.9%	14.3%
MH Only	0.0%	0.0%	2.2%	4.3%	6.7%	8.9%	11.1%	13.2%
SU Only	0.0%	0.0%	2.9%	5.7%	9.1%	12.6%	15.5%	18.1%
Both MH and SU	0.0%	0.0%	2.4%	5.1%	7.3%	9.8%	12.3%	14.9%

Years Since New Diabetes Diagnosis

© 2019 Schmidt EM et al. *JAMA Network Open.*

Sensitivity Analysis 1: Missingness of Outcome Data Points Among Patients Who Were Alive into Year 7

eFigure 3 displays missingness in the sensitivity analysis limited to patients who were alive into year 7 (see also eTable 6 in the Supplement). That is, missingness in eFigure 3 represents the proportion of patients who did not use VHA care during a given year, among patients who remained alive throughout the study. Patients in the No MH or SU group had higher rates of missingness due to not using VHA care during every time period, compare to patients in any MH or SU group.

eFigure 3. Missingness for Outcome Data Points Due to Not Using VHA Health Care in a Given Time Period, Among Patients Who Were Alive Into Year 7 After Diabetes Onset (Sensitivity Analysis 1)

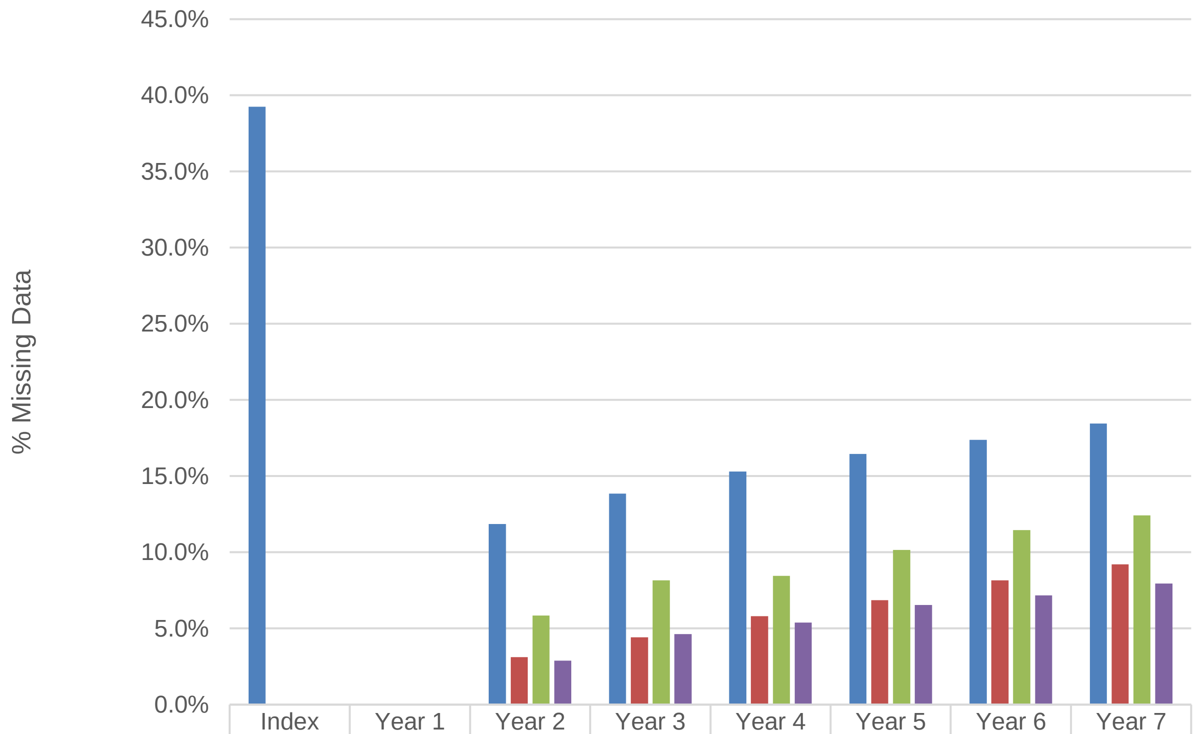

	Index	Year 1	Year 2	Year 3	Year 4	Year 5	Year 6	Year 7
No MH or SU	39.2%	0.0%	11.9%	13.9%	15.3%	16.5%	17.4%	18.4%
MH Only	0.0%	0.0%	3.1%	4.4%	5.8%	6.8%	8.2%	9.2%
SU Only	0.0%	0.0%	5.8%	8.2%	8.5%	10.2%	11.5%	12.4%
Both MH and SU	0.0%	0.0%	2.9%	4.6%	5.4%	6.5%	7.2%	8.0%

Years Since New Diabetes Diagnosis

© 2019 Schmidt EM et al. *JAMA Network Open.*

Sensitivity Analysis 2: Missingness Among Patients Who Made at Least 2 Visits to Primary Care before their New Diabetes Diagnosis was Given

eFigure 4 displays missingness in the sensitivity analysis that only included patients who made at least 2 visits to primary care in the 2-year period preceding their new diabetes diagnosis (see also eTable 6 in this Supplement). In Sensitivity Analysis 2, patients in any MH or SU group high the highest rates of missingness, compared to patients in the No MH or SU group.

eFigure 4. Missingness for Outcome Data Points Due to Mortality or to Not Using VHA Health Care in a Given Time Period, Among Patients Who Made at Least 2 Visits to Primary Care Before New Onset Diabetes (Sensitivity Analysis 2)

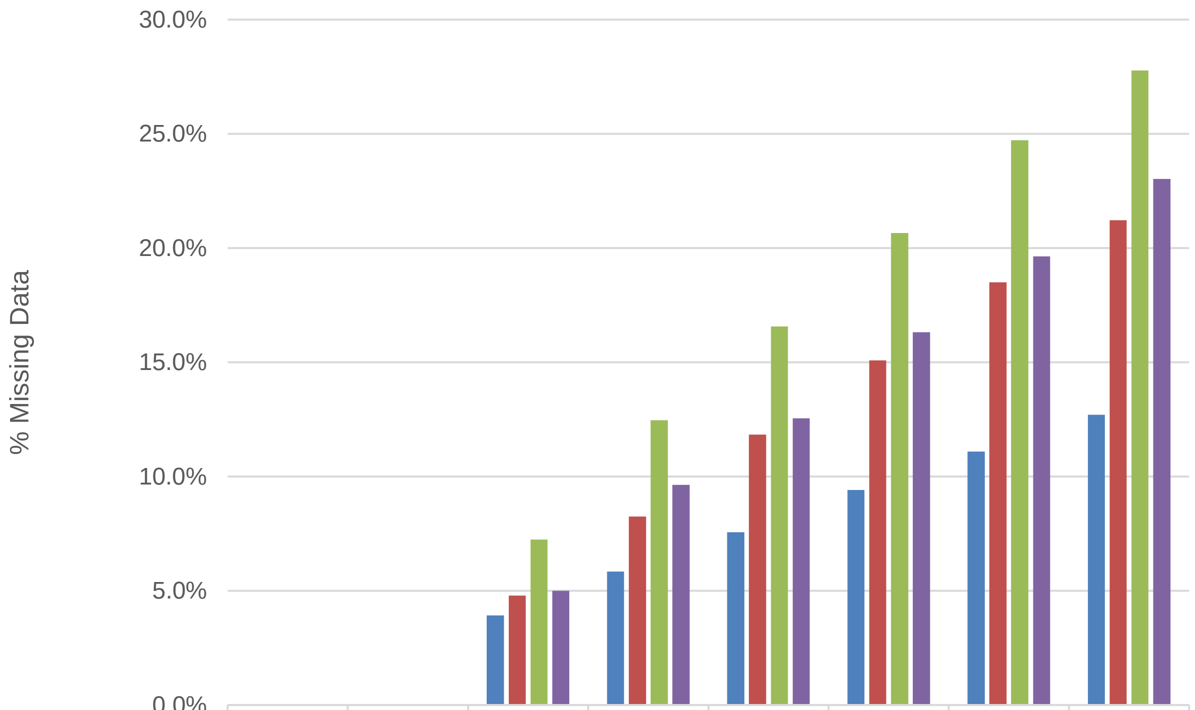

	Index	Year 1	Year 2	Year 3	Year 4	Year 5	Year 6	Year 7
No MH or SU	0.0%	0.0%	3.9%	5.8%	7.6%	9.4%	11.1%	12.7%
MH Only	0.0%	0.0%	4.8%	8.3%	11.8%	15.1%	18.5%	21.2%
SU Only	0.0%	0.0%	7.2%	12.5%	16.6%	20.7%	24.7%	27.8%
Both MH and SU	0.0%	0.0%	5.0%	9.6%	12.6%	16.3%	19.6%	23.0%

Years Since New Diabetes Diagnosis

© 2019 Schmidt EM et al. *JAMA Network Open.*

Number of Outcome Data Points Analyzed per Patient (i.e., "Completeness" of Outcome Data)

The level of missing outcome data in our analysis should be interpreted in the context of the level of each patient's data completeness as well. We used mixed-effects linear regression to run a longitudinal growth model with 8 total time points during which the outcome variable was calculated. That is, each patient's medical record data could lead to a maximum of 8 outcome data points observed over time in our analyses. Among the cohort as a whole, a technical majority of patients had at least 1 missing outcome data point at during a time period in our analyses (median outcome data points per patients = 7). However, for the main analysis, an average of 6.5 (*SD* = 2.1) outcome data points were analyzed per patient, indicating that most patients had many outcome data points over time, even if that patient had missing data during any given year or years.

eTable 1 displays a percent distribution for the number of outcome values analyzed per patient, overall and by comparative MHSU groups. Consistent with figures above, there was a higher proportion of patients in the No MH or SU disorder group with 7 rather than 8 outcome values observed, notably as a result of no health care utilization prior to receiving their first diabetes diagnosis.

eTable 1. Percent Distribution of Number of Outcome Data Points Analyzed per Patient Across Eight Total Time Periods Possible

Number of Outcome Data Points Analyzed	Mental Health Disorder Only (n = 20,032)	Substance Use Disorder Only (n = 2,800)	Mental Health and Substance Use Disorder (n = 5,801)	No Mental or Substance Use Disorder (n = 94,359)	Total (N = 122,992)
1 Data Point (minimum possible) (%)	0.0	0.0	0.0	4.6	3.5
2 Data Points (%)	3.6	4.9	3.1	6.4	5.8
3 Data Points (%)	3.6	5.2	3.9	5.4	5.0
4 Data Points (%)	3.9	5.5	3.8	5.2	5.0
5 Data Points (%)	3.8	5.7	4.3	5.8	5.4
6 Data Points (%)	4.6	6.0	4.8	7.2	6.7
7 Data Points (%)	6.1	9.0	6.9	26.3	21.7
8 Data Points (maximum possible) (%)	74.4	63.9	73.2	39.2	47.1

© 2019 Schmidt EM et al. *JAMA Network Open.*

Rates of Specific Mental Health or Substance Use Disorder Diagnoses Before Diabetes Onset

This section of supplemental material contains percentages of specific mental health (MH) or substance use (SU) disorder diagnoses made in the Index period of analyses. That is, MH or SU diagnoses displayed in eTable 2 were assigned by a provider in the 2-year period prior to the date a patient received his or her new diabetes diagnosis. Some patients had multiple diagnoses for MH disorders, SU disorders, or both, so column totals may not equal 100%. Note also, in eTable 2, we do not display a column for patients in the No MH or SU disorder group because, by definition, this group received zero MHSU diagnoses. Cells are left blank for not-applicable combinations of MH or SU group membership and specific diagnosis groups (e.g., Major Depressive Disorder for the SU Only group).

eTable 2. Percent of Patients with Pre-Existing Mental Health or Substance Use Disorder Diagnoses

Diagnosis Group	Mental Health Disorder Only (n = 20,032)	Substance Use Disorder Only (n = 2,800)	Mental Health and Substance Use Disorder (n = 5,801)	Total Cohort (N = 122,992)
Mental Health Disorders				
Major Depressive Disorders (%)	19.2		27.9	4.4
Other Depressive Disorders(%)	57.2		64.1	12.4
Affective and Bipolar Disorders (%)	7.3		15.0	1.9
Schizophrenia (%)	7.0		11.7	1.7
Other Psychoses (%)	3.7		7.9	
Posttraumatic Stress Disorders (%)	36.9		44.0	8.1
Generalized Anxiety Disorders (%)	5.6		5.9	1.2
Other Anxiety Disorders (%)	20.2		19.9	4.2
Personality Disorders (%)	3.0		8.6	0.9
Substance Use Disorders				
Alcohol Use Disorders (%)		78.4	79.5	5.5
Cocaine Use Disorders (%)		16.8	24.4	1.5
Other Simulant Use Disorders (Amphetamines) (%)		1.3	3.0	0.2
Cannabis Use Disorders (%)		6.6	15.9	0.9
Opioid Use Disorders (%)		7.1	10.6	0.7
Barbiturate Use Disorders (%)		0.5	2.2	0.1

© 2019 Schmidt EM et al. *JAMA Network Open.*

Rates of Specific Diabetes Complications and Severity Over Time

This section of supplemental material first provides illustrative examples of the specific diagnoses for diabetes complications that were grouped, and weighted, to calculate a patient's score on the Diabetes Complication Severity Index (DCSI). Note that the method for calculating the DCSI was developed and described fully by Young et al.[1] Briefly, we analyzed patient's medical record to identify each diagnosis in the DCSI representing a medical abnormality consistent with diabetes complications (e.g., diabetic ophthalmologic disease). Each diagnosis we identified was assigned a weight of 1 or 2, per Young et al.'s specifications.[1] Within a category of complications such as Retinopathy or Nephropathy (i.e., the italicized rows in eTable3), the sum of weighted scores was calculated for a within-category total. The maximum within-category total allowed was 2 points, except for the neuropathy category which was only weighted at 1 point maximum (per Young et al.'s specification[1]). The within-category totals were then summed across categories to calculate a patient's total DCSI score, for a possible range of 0 to 13 points. Please consult Young and colleagues' paper for full details about calculating the DCSI.[1]

eTable 3. Example Diagnoses Included in the Diabetes Complication Severity Index, by Category of Complications

**Category of Complications (weighted score) in the Diabetes Complication Severity Index**	**Illustrative Examples of Diagnoses Included in Group**
Retinopathy	
Mild Retinopathy (weighted score = 1)	Diabetic ophthalmologic disease<br>Background retinopathy<br>Retinal edema
Severe Retinopathy (weighted score = 2)	Proliferative retinopathy<br>Retinal detachment<br>Blindness
Nephropathy	
Mild Nephropathy (weighted score = 1)	Diabetic nephropathy<br>Acute glomerulonephritis<br>Nephritis/nephropathy
Severe Nephropathy (weighted score = 2)	Chronic renal failure<br>Renal insufficiency
Neuropathy	
Neuropathy (weighted score = 1 for all)	Diabetic neuropathy<br>Mononeuropathy<br>Gastroparesis
Cerebrovascular	
Mild Cerebrovascular (weighted score = 1)	Transient Ischemic Attack
Severe Cerebrovascular (weighted score = 2)	Stroke
Cardiovascular	
Mild Cardiovascular (weighted score = 1)	Atherosclerosis<br>Other IHD<br>Angina pectoris
Severe Cardiovascular (weighted score = 2)	Myocardial infarction<br>Ventricular fibrillation, arrest<br>Heart failure
Peripheral Vascular Disease	
Mild Peripheral Vascular Disease (weighted score = 1)	Diabetic PVD<br>Other aneurysm, Lower extremity<br>Foot wound with complication
Severe Peripheral Vascular Disease (weighted score = 2)	Gangrene<br>Gas Gangrene<br>Ulcer of lower limbs

© 2019 Schmidt EM et al. *JAMA Network Open.*

**Category of Complications (weighted score) in the Diabetes Complication Severity Index**	**Illustrative Examples of Diagnoses Included in Group**
Metabolic Complications	
Metabolic Complications (weighted score = 2 for all)	Ketoacidosis<br>Hyperosmolar<br>Other coma

© 2019 Schmidt EM et al. *JAMA Network Open.*

eTable 4 contains the percent of patients who received a diagnosis for for each category of complications in the DCSI, by comparative MH or SU disorder groups. In a given time period, each patient could have had both a mild and a severe complication within a given category. As such, column totals in eTable 4 may not total to 100%, nor will a row total to the “any” complication data presented in Table 2 of the manuscript. Table 2 of the manuscript displays rates of patients' highest-weighted within-category score on the DCSI across all 8 time periods. In eTable 4 of the Supplement, we present full data on event rates for the outcome variable, by comparative group, and by the 8 time periods analyzed in mixed-effects regressions.

Note also that we provide percentages in eTable 4 based on the number of patients who were alive in each time period (denominators are provided in the top rows of each respective section of the table).

eTable 4. Percent of Patients Diagnosed With a Specific Category of Diabetes Complications, by Severity of Complication, by Time Period, and by Comparative MHSU Groups

Total Cohort								
**Group of Complications**	**Index (n=122992)**	**Year 1 (n=122992)**	**Year 2 (n=119991)**	**Year 3 (n=117056)**	**Year 4 (n=114159)**	**Year 5 (n=111290)**	**Year 6 (n=108377)**	**Year 7 (n=105466)**
Mild Retinopathy	0.9%	8.7%	6.5%	6.7%	6.7%	6.8%	7.1%	7.1%
Severe Retinopathy	1.9%	3.6%	2.8%	3.0%	3.1%	3.4%	3.4%	3.5%
Mild Nephropathy	0.1%	1.8%	1.6%	1.8%	2.0%	2.1%	2.3%	2.4%
Severe Nephropathy	1.0%	5.4%	5.3%	5.9%	6.7%	7.3%	8.0%	8.9%
Neuropathy	5.7%	14.4%	12.7%	13.3%	14.1%	14.8%	16.0%	17.2%
Mild Cerebrovascular	0.8%	1.1%	0.9%	0.8%	0.9%	0.9%	0.9%	0.9%
Severe Cerebrovascular	3.0%	5.4%	4.5%	4.5%	4.6%	4.8%	4.9%	5.1%
Mild Cardiovascular	14.7%	25.6%	21.4%	21.1%	21.2%	21.2%	21.0%	21.3%
Severe Cardiovascular	7.6%	13.2%	11.0%	11.3%	11.7%	12.0%	12.3%	12.9%
Mild Peripheral Vascular Disease	2.9%	5.7%	4.6%	4.6%	4.7%	4.9%	4.9%	5.2%
Severe Peripheral	0.8%	1.7%	1.3%	1.4%	1.4%	1.6%	1.7%	2.0%

© 2019 Schmidt EM et al. *JAMA Network Open.*

Vascular Disease								
Metabolic Complications	0.0%	0.4%	0.2%	0.2%	0.2%	0.2%	0.2%	0.2%
No Mental Health or Substance Use Disorder								
**Group of Complications**	**Index (n=94359)**	**Year 1 (n=94359)**	**Year 2 (n=92012)**	**Year 3 (n=89743)**	**Year 4 (n=87551)**	**Year 5 (n=85368)**	**Year 6 (n=83124)**	**Year 7 (n=80845)**
Mild Retinopathy	0.7%	9.7%	7.3%	7.5%	7.5%	7.6%	7.9%	7.9%
Severe Retinopathy	1.5%	3.7%	2.9%	3.1%	3.2%	3.5%	3.5%	3.5%
Mild Nephropathy	0.1%	2.1%	1.8%	2.0%	2.2%	2.3%	2.5%	2.7%
Severe Nephropathy	0.8%	6.0%	5.8%	6.4%	7.3%	7.9%	8.6%	9.5%
Neuropathy	3.6%	14.0%	12.0%	12.8%	13.5%	14.3%	15.3%	16.5%
Mild Cerebrovascular	0.6%	1.1%	0.8%	0.8%	0.9%	0.8%	0.8%	0.9%
Severe Cerebrovascular	2.3%	5.4%	4.4%	4.3%	4.5%	4.7%	4.8%	5.0%
Mild Cardiovascular	12.6%	26.7%	21.7%	21.4%	21.4%	21.4%	21.3%	21.5%
Severe Cardiovascular	6.5%	13.7%	11.0%	11.4%	11.8%	12.1%	12.4%	13.0%
Mild Peripheral Vascular Disease	2.3%	5.8%	4.6%	4.7%	4.7%	4.9%	5.0%	5.3%
Severe Peripheral Vascular Disease	0.5%	1.7%	1.4%	1.5%	1.5%	1.6%	1.8%	2.0%
Metabolic Complications	0.0%	0.4%	0.2%	0.1%	0.1%	0.2%	0.2%	0.2%
Mental Health Disorder Only								
**Group of Complications**	**Index (n=20032)**	**Year 1 (n=20032)**	**Year 2 (n=19600)**	**Year 3 (n=19170)**	**Year 4 (n=18686)**	**Year 5 (n=18240)**	**Year 6 (n=17801)**	**Year 7 (n=17388)**
Mild Retinopathy	1.5%	5.2%	3.7%	4.0%	4.0%	4.0%	4.3%	4.5%

© 2019 Schmidt EM et al. *JAMA Network Open.*

Severe Retinopathy	3.1%	3.1%	2.6%	2.9%	2.7%	3.0%	3.2%	3.2%
Mild Nephropathy	0.1%	1.0%	1.0%	1.1%	1.3%	1.4%	1.4%	1.7%
Severe Nephropathy	1.4%	3.5%	3.8%	4.6%	5.3%	5.7%	6.2%	7.1%
Neuropathy	12.9%	16.1%	14.9%	15.4%	16.2%	17.0%	18.5%	19.9%
Mild Cerebrovascular	1.7%	1.3%	1.2%	1.1%	1.3%	1.3%	1.2%	1.1%
Severe Cerebrovascular	5.5%	5.4%	5.0%	5.1%	5.2%	5.3%	5.4%	5.3%
Mild Cardiovascular	23.9%	24.3%	22.4%	21.9%	22.3%	22.1%	21.6%	22.1%
Severe Cardiovascular	11.7%	12.0%	11.2%	11.5%	11.6%	12.0%	12.1%	12.8%
Mild Peripheral Vascular Disease	4.5%	5.0%	4.1%	4.0%	4.0%	4.3%	4.3%	4.5%
Severe Peripheral Vascular Disease	1.3%	1.4%	1.1%	1.1%	1.1%	1.2%	1.3%	1.5%
Metabolic Complications	0.0%	0.5%	0.1%	0.1%	0.1%	0.1%	0.1%	0.2%
Substance Use Disorder Only								
**Group of Complications**	**Index (n=2800)**	**Year 1 (n=2800)**	**Year 2 (n=2719)**	**Year 3 (n=2640)**	**Year 4 (n=2544)**	**Year 5 (n=2447)**	**Year 6 (n=2367)**	**Year 7 (n=2294)**
Mild Retinopathy	1.9%	7.0%	5.3%	5.3%	5.7%	5.8%	5.9%	6.1%
Severe Retinopathy	2.6%	3.0%	2.5%	2.4%	2.6%	2.8%	3.0%	3.9%
Mild Nephropathy	0.1%	0.8%	0.8%	0.9%	1.4%	1.2%	1.4%	1.3%
Severe Nephropathy	1.4%	3.2%	3.4%	4.2%	4.2%	5.3%	5.7%	6.7%
Neuropathy	9.1%	13.8%	11.5%	12.5%	14.0%	15.0%	16.0%	17.0%
Mild Cerebrovascular	0.9%	0.8%	0.7%	0.6%	0.7%	0.7%	0.7%	0.6%

© 2019 Schmidt EM et al. *JAMA Network Open.*

Severe Cerebrovascular	5.6%	5.7%	5.1%	4.8%	4.8%	5.1%	5.5%	5.9%
Mild Cardiovascular	15.6%	17.2%	15.7%	15.9%	15.6%	16.3%	17.0%	17.3%
Severe Cardiovascular	10.7%	12.4%	11.1%	11.1%	12.0%	12.2%	12.6%	12.7%
Mild Peripheral Vascular Disease	5.8%	7.1%	6.6%	5.8%	6.5%	7.0%	6.1%	6.9%
Severe Peripheral Vascular Disease	2.6%	3.1%	1.8%	2.2%	2.2%	2.5%	2.2%	2.9%
Metabolic Complications	0.0%	1.5%	0.4%	0.4%	0.4%	0.3%	0.4%	0.5%
Both Mental Health and Substance Use Disorder								
**Group of Complications**	**Index (n=**5801**)**	**Year 1 (n=**5801**)**	**Year 2 (n=**5660**)**	**Year 3 (n=**5503**)**	**Year 4 (n=**5378**)**	**Year 5 (n=**5235**)**	**Year 6 (n=**5085**)**	**Year 7 (n=**4939**)**
Mild Retinopathy	1.9%	5.9%	4.2%	4.0%	3.9%	3.8%	4.2%	4.1%
Severe Retinopathy	3.8%	3.3%	2.7%	2.6%	2.5%	2.8%	2.8%	2.8%
Mild Nephropathy	0.2%	0.9%	0.9%	1.0%	1.2%	1.4%	1.7%	1.5%
Severe Nephropathy	1.4%	3.0%	3.4%	3.5%	4.3%	4.7%	5.5%	6.5%
Neuropathy	13.5%	16.2%	15.4%	15.1%	15.6%	16.4%	17.5%	19.0%
Mild Cerebrovascular	1.4%	1.1%	0.9%	0.8%	0.7%	0.9%	0.9%	0.7%
Severe Cerebrovascular	4.8%	4.6%	4.3%	4.2%	4.3%	4.9%	5.0%	5.1%
Mild Cardiovascular	17.6%	17.7%	16.6%	16.4%	16.6%	16.7%	16.4%	17.3%
Severe Cardiovascular	10.0%	10.3%	9.7%	10.0%	10.4%	10.2%	11.1%	11.9%
Mild Peripheral Vascular Disease	5.2%	5.7%	5.1%	5.3%	5.1%	5.4%	5.1%	5.3%

© 2019 Schmidt EM et al. *JAMA Network Open.*

Severe Peripheral Vascular Disease	1.9%	2.1%	1.4%	2.1%	1.3%	1.5%	1.8%	1.9%
Metabolic Complications	0.0%	1.0%	0.2%	0.4%	0.4%	0.4%	0.3%	0.4%

© 2019 Schmidt EM et al. *JAMA Network Open.*

Complete Mixed-Effects Regression Analysis Results

This section of the Supplement provides complete mixed-effects regression results for the main analysis and for both sensitivity analyses.

eTable 5. Complete Mixed Effects Regression Model Results With All Covariates (See also Table 3 in Manuscript)

Variable [Reference Category]	Model A, Unconditional Growth Model		Model B, Conditional, Unadjusted		Model C, Fully Adjusted	
	**Est. (SE)**	***p***	**Est. (SE)**	***p***	**Est. (SE)**	***p***
**Initial DCSI Score**						
Intercept	.845 (.010)	<.001	.841 (.011)	<.001	-.310 (.023)	<.001
Mental or Substance Use Disorder [ref: None]						
MH Only			.010 (.010)	.32	.015 (.010)	.12
SU Only			-.081 (.024)	.001	-.088 (.023)	<.001
MH and SU			-.041 (.017)	.01	-.126 (.017)	<.001
Age in years					.023 (.0002)	<.001
Male [ref: Female]					.237 (.017)	<.001
Race/Ethnicity [ref: White, non-Hispanic]						
Black					-.052 (.009)	<.001
Hispanic					-.094 (.014)	<.001
Asian/Pacific Islander					-.126 (.024)	<.001
American Indian/ Alaska Native					-.049 (.033)	.144
Married [ref: Not Married]					.052 (.006)	<.001
Homeless [ref: Not Homeless]					.030 (.017)	.08
Rurality [ref: Urban]						
Rural					-.013 (.007)	.07
Highly Rural					-.070 (.028)	.01
Medical Comorbidities [dummy coded]						
Pulmonary Circulation Disorder					.485 (.050)	<.001
Hypertension					.102 (.008)	<.001
Paralysis					.489 (.038)	<.001
Other Neurological Disorder					.174 (.021)	<.001
Chronic Obstructive Pulmonary Disease					.141 (.010)	<.001
Hypothyroidism					.024 (.016)	.13
Liver Disease					-.152 (.021)	<.001
Chronic Peptic Ulcer					.022 (.029)	.45
Solid Tumor w/o Metastases					-.051 (.012)	<.001
Rheumatoid Arthritis					.065 (.027)	.02

© 2019 Schmidt EM et al. *JAMA Network Open.*

Variable [Reference Category]	Model A, Unconditional Growth Model		Model B, Conditional, Unadjusted		Model C, Fully Adjusted	
	**Est. (SE)**	***p***	**Est. (SE)**	***p***	**Est. (SE)**	***p***
Coagulopathy					.682 (.027)	<.001
Fluid and Electrolyte Disorders					.162 (.021)	<.001
Blood Loss Anemia					.220 (.123)	.07
Deficiency Anemia					.191 (.191)	<.001
Obesity					-.043 (.008)	<.001
Tobacco Use Disorder					.093 (.010)	<.001
Inpatient Index Diabetes Diagnosis [ref: Outpatient]					.448 (.015)	<.001
Primary Care Visits [ref: 0 Visits]						
1-2 visits					-.408 (.009)	<.001
3-4 visits					-.497 (.011)	<.001
5-8 visits					-.387 (.011)	<.001
9+ visits					-.149 (.013)	<.001
Medical Inpatient Stay [ref: No Stay]					.402 (.012)	<.001
DCSI Progression Over Time						
Time in years	.083 (.002)	<.001	.110 (.003)	<.001	.102 (.003)	<.001
$Time^2$ in years	-.002 (.0003)	<.001	-.004 (.0003)	<.001	-.0034 (.0003)	<.001
Interaction: Time X MH or SU Status [ref: no MH or SU]						
MH Only			-.076 (.005)	<.001	-.070 (.005)	<.001
SU Only			-.048 (.012)	<.001	-.042 (.012)	<.001
MH and SU			-.100 (.008)	<.001	-.093 (.008)	<.001
Interaction: $Time^2$ X MH or SU Status [ref: no MH or SUD]						
MH Only			.007 (.0006)	<.001	.0064 (.0006)	<.001
SU Only			.005 (.0016)	.001	.0045 (.0016)	.005
MH and SU			.009 (.0011)	<.001	.0084 (.0011)	<.001
Variance Components						
Level 1 Residual	.924		.925		.931	
Level 2						
Intercept	.897		.897		.774	
Time	.125		.124		.120	
Correlation	.172		.171		.307	
Level 3						
Intercept	.105		.105		.083	
Time	.014		.014		.014	
Correlation	.072		.079		.064	
Fit Statistics						
Deviance	-1179763		-1179492		-1170937	
AIC	2359548		2359018		2341979	
BIC	2359676		2359215		2342581	

© 2019 Schmidt EM et al. *JAMA Network Open.*

eTable 6. Complete Mixed Effects Regression Model Results From Sensitivity Analyses

Variable [Reference Category]	Sensitivity Analysis: Exclude Ps without Primary Care Use (N = 102,489)		Sensitivity Analysis: Exclude Ps who Died during Observation Period (N = 66,834)	
	**Estimate (SE)**	***p***	**Estimate (SE)**	***p***
Intercept	-.223 (.022)	<.001	-.810 (.026)	<.001
Mental or Substance Use Disorder [ref: None]				
MH Only	.017 (.010)	.09	.053 (.010)	<.001
SU Only	-.063 (.024)	.009	.007 (.025)	.76
MH and SU	-.097 (.018)	<.001	-.085 (.017)	<.001
Age in years	.020 (.0003)	<.001	.026 (.0004)	<.001
Male [ref: Female]	.218 (.016)	<.001	.216 (.019)	<.001
Race/Ethnicity [ref: White, non-Hispanic]				
Black	-.053 (.009)	<.001	-.054 (.010)	<.001
Hispanic	-.079 (.014)	<.001	-.163 (.017)	<.001
Asian/Pacific Islander	-.120 (.024)	<.001	-.130 (.030)	<.001
American Indian/ Alaska Native	-.054 (.034)	.11	-.073 (.045)	.11
Married [ref: Not Married]	.041 (.006)	<.001	.010 (.008)	.19
Homeless [ref: Not Homeless]	-.010 (.018)	.56	.063 (.018)	.001
Rurality [ref: Urban]				
Rural	-.007 (.007)	.31	-.001 (.008)	.88
Highly Rural	-.024 (.028)	.40	-.048 (.031)	.12
Medical Comorbidities [dummy coded]				
Pulmonary Circulation Disorder	.418 (.060)	<.001	.488 (.048)	<.001
Hypertension	.100 (.008)	<.001	.135 (.008)	<.001
Paralysis	.534 (.041)	<.001	.528 (.038)	<.001
Other Neurological Disorder	.160 (.023)	<.001	.185 (.020)	<.001
Chronic Obstructive Pulmonary Disease	.122 (.010)	<.001	.147 (.009)	<.001
Hypothyroidism	.031 (.016)	.05	.019 (.015)	.20
Liver Disease	-.121 (.022)	<.001	-.138 (.021)	<.001
Chronic Peptic Ulcer	.066 (.030)	.03	.024 (.027)	.38
Solid Tumor w/o Metastases	-.044 (.013)	.001	-.049 (.012)	<.001
Rheumatoid Arthritis	.041 (.028)	.15	.065 (.026)	.01
Coagulopathy	.684 (.030)	<.001	.683 (.026)	<.001
Fluid and Electrolyte Disorders	.139 (.022)	<.001	.157 (.020)	<.001
Blood Loss Anemia	.137 (.133)	.30	.264 (.115)	.02

© 2019 Schmidt EM et al. *JAMA Network Open.*

Variable [Reference Category]	Sensitivity Analysis: Exclude Ps without Primary Care Use (N = 102,489)		Sensitivity Analysis: Exclude Ps who Died during Observation Period (N = 66,834)	
	**Estimate (SE)**	***p***	**Estimate (SE)**	***p***
Deficiency Anemia	.148 (.017)	<.001	.182 (.015)	<.001
Obesity	-.041 (.008)	<.001	-.022 (.008)	.007
Tobacco Use Disorder	.084 (.010)	<.001	.117 (.009)	<.001
Inpatient Index Diabetes Diagnosis [ref: Outpatient]	.437 (.016)	<.001	.327 (.018)	<.001
Primary Care Visits [ref: 0 Visits]			Excluded	
1-2 visits	-.347 (.009)	<.001	Reference Category	
3-4 visits	-.425 (.011)	<.001	-.078 (.012)	<.001
5-8 visits	-.324 (.011)	<.001	.020 (.012)	.09
9+ visits	-.109 (.013)	<.001	.238 (.013)	<.001
Medical Inpatient Stay [ref: No Stay]	.355 (.013)	<.001	.420 (.012)	<.001
Time in years	.090 (.003)	<.001	.062 (.003)	<.001
$Time^2$ in years	-.0016 (.0003)	<.001	.0005 (.0004)	.23
Interaction: Time X MH or SU Status [ref: no MH or SU]				
MH Only	-.058 (.005)	<.001	-.053 (.005)	<.001
SU Only	-.039 (.012)	.001	-.047 (.012)	<.001
MH and SU	-.087 (.008)	<.001	-.067 (.008)	<.001
Interaction: $Time^2$ X MH or SUH Status [ref: no MH or SUD]				
MH Only	.0049 (.0006)	<.001	.0053 (.0007)	<.001
SU Only	.0045 (.0016)	.006	.0058 (.0017)	.001
MH and SU	.0075 (.0011)	<.001	.0060 (.0011)	<.001
Variance Components				
Level 1 Residual	.882		.871	
Level 2				
Intercept	.707		.751	
Time	.123		.116	
Correlation	.259		.118	
Level 3				
Intercept	.079		.0003	
Time	.014		.001	
Correlation	-.036		.012	
Fit Statistics				
Deviance	-1004397		-666419	
AIC	2008897		1332939	
BIC	2009493		1333504	

© 2019 Schmidt EM et al. *JAMA Network Open.*

eReference

1. Young BA, Lin E, Von Korff M, et al. Diabetes complications severity index and risk of mortality, hospitalization, and healthcare utilization. *The American journal of managed care.* 2008;14(1):15-23.

© 2019 Schmidt EM et al. *JAMA Network Open.*
